# Clinical Efficacy and Safety Profile of Prucalopride in Chronic Idiopathic Constipation

**DOI:** 10.7759/cureus.4382

**Published:** 2019-04-04

**Authors:** Asim Tameez Ud Din, Ameer H Khan, Hamza Bajwa, Muhammad Haisum Maqsood, Mustafa N Malik

**Affiliations:** 1 Internal Medicine, Rawalpindi Medical College, Rawalpindi, PAK; 2 Internal Medicine, Allama Iqbal Medical College, Lahore, PAK; 3 Internal Medicine, King Edward Medical University / Mayo Hospital, Lahore, PAK; 4 Internal Medicine, The University of Arizona, Tucson, USA

**Keywords:** prucalopride, chronic idiopathic constipation, constipation, clinical efficacy of prucalopride, safety profile of prucalopride

## Abstract

Chronic idiopathic constipation (CIC) can be defined as bowel movements that are difficult to pass, are not occurring frequently, or have incomplete evacuation during defecation. A high-fiber diet and laxatives are the commonly used treatments, but in many cases, they do not produce satisfactory results. The first line of treatment is osmotic laxatives. If there is no improvement, the second line is guanylate cyclase-C (GCC) agonists like linaclotide or prokinetic agents such as prucalopride. On December 14, 2018, the United States Food and Drug Administration (US FDA) approved prucalopride for treating chronic idiopathic constipation. Prucalopride is a prokinetic agent which works at the 5-hydroxytryptamine receptor 4 (5-HT4) as an agonist with greater receptor selectivity. Patients on prucalopride reported improved symptoms, quality of life and satisfaction. The most frequent adverse events were headaches and problems related to the gastrointestinal tract. Caution should be taken when using prucalopride in patients with impaired liver and renal function. In Canada, prucalopride has been approved for treatment of female patients with chronic idiopathic constipation who have failed therapy with at least two laxatives from different classes over a six-month period.

## Introduction and background

Chronic idiopathic constipation (CIC) can be defined as bowel movements that are difficult to pass, are not occurring frequently, or have incomplete evacuation during defecation. According to the Rome IV criteria, constipation should be present for three months with the onset of symptoms at least six months before for making a diagnosis [1]. It should also include two or more of the following symptoms in more than one-fourth of defecations: straining, hard stools, the sensation of anorectal obstruction, incomplete evacuation, use of manual maneuvers for bowel evacuation or less than three spontaneous bowel movements in a week. Furthermore, irritable bowel syndrome should be ruled out and there should be rare occurrence of loose stools without laxative use. There are three types of chronic idiopathic constipation. The first type is normal-transit constipation (stool takes one and a half to three days to pass through the colon) [2]. Second is slow-transit constipation (stool takes more than five days to pass through the colon, also called colonoparesis) [3]. Third is outlet dysfunction (it is associated with a sense of incomplete evacuation) [4]. Everhart et al. concluded a positive relationship between constipation and low socioeconomic status [5]. This condition can significantly impact a person's quality of life and productivity. In the majority of cases, the condition is initially treated empirically without investigation for a cause. High-fiber diet and laxatives are the commonly used treatments, but in many cases, they do not produce satisfactory results [6]. These patients are then treated by other medications, behavioral therapy, or surgical intervention. In CIC with slow transit, the first line of treatment is osmotic laxatives. If there is no improvement, the second line is guanylate cyclase-C (GCC) agonists like linaclotide or prokinetic agents such as prucalopride [7]. On December 14, 2018, the United States Food and Drug Administration (US FDA) approved prucalopride for treating chronic idiopathic constipation [8]. The aim of our study is to review the published literature on the clinical efficacy and safety of prucalopride in treating chronic idiopathic constipation.

## Review

Mechanism of action

Prucalopride is a prokinetic agent which works at the 5-hydroxytryptamine receptor 4 (5-HT4) as an agonist with greater receptor selectivity and less proarrhythmic risk as compared to other members of its class [9]. The role of prucalopride in improving constipation is attributed to its ability to increase the number of synchronous contractions in the large intestine and simultaneously decrease the frequency of isolated contractions in the proximal colon [10]. It stimulates giant migratory contractions in the colon, the lack of which is a possible underlying mechanism for CIC. Increasing gastrointestinal motility is especially beneficial for patients of chronic constipation who lack these high amplitude propagating contractions (HAPS). These prokinetic effects are not limited to the large bowel. It also accelerates gastric emptying and small bowel transit [11].

Recommended Dose

It is generally given at a dose of 2-4 mg/day in patients with CIC. However, the recommended dose for elderly patients is 1 mg [12].

Clinical efficacy and quality of life

Patients on prucalopride reported improved symptoms, quality of life, and satisfaction. In a phase III study by Quigley et al. including 641 patients with severe chronic constipation, 24% patients taking prucalopride as compared to 12% in the placebo group achieved three or more spontaneous complete bowel movements (SCBMs) per week [13].

Camilleri et al. studied the efficacy of prucalopride in patients with chronic constipation. Patients from three double-blind, placebo-controlled, 12-week studies with prucalopride were allowed to continue treatment in open-label studies up to 24 months. Out of the 1,455 patients who completed the primary studies, 86% continued with the prucalopride. The improvement in the Patient Assessment of Constipation Quality of Life questionnaire (PAC-QOL) satisfaction score seen after a 12-week treatment was preserved in the open-label treatment for up to 18 months [14].

In a randomized, double-blind, phase 3 study by Yiannakou et al. including 374 patients, 142 (37.9%) patients achieved a mean of three or more SCBMs per week in the prucalopride group as compared to 67 (17.7%) patients in the placebo group (p <0.0001). The proportion of patients with an improvement of at least one point in Patient Assessment of Constipation Quality of Life questionnaire (PAC-QOL) satisfaction subscale score was 52.7% and 38.8% in the prucalopride and placebo groups, respectively (p = 0.0035) [15].

An integrated analysis of six randomized clinical trials was done by Camilleri et al to determine the efficacy of prucalopride in patients with chronic constipation. A total of 2,484 patients including 597 men and 1,887 women were included in the study. Of this, 27.8% in the prucalopride group as compared to 13.2% in the placebo group when treated over 12 weeks achieved a mean of ≥3 SCBMs/week (p < 0.001) [16].

Safety profile

In a study by Camilleri et al. including 1,455 patients, the most frequent adverse events were headaches (1%) and problems related tothe gastrointestinal tract (3.3%) [14]. Another trial to determine the safety of prucalopride in older patients with chronic constipation who are living in nursing homes was done. Most of the patients included in this study had a history of cardiovascular disease. Only one female who had a history of pacemaker and heart disease showed prolongation of QT interval from baseline [17]. Caution should be taken when using prucalopride in patients with impaired liver function and renal function. Since it is excreted in the urine unchanged, the dose is reduced to 1 mg once daily in patients with impaired renal function [18]. It is also not recommended for use during pregnancy or lactation (category C drug) [19].

Chronic Constipation Diagnosis and Treatment Evaluation: (CHRO.CO.DI.T.E. Study)

Bellini et al. focused on the different diagnostic and therapeutic options considered by Italian gastroenterologists in patients with chronic constipation, especially constipation-predominant irritable bowel syndrome. Most of them used Macrogol, a laxative, as the first line of treatment along with lifestyle modifications and an increased fiber diet. Prucalopride was used mainly as a second or third line of treatment due to its high cost [20].

Other clinical implications of prucalopride

Prucalopride can be used for treating constipation in patients with chronic opioid use and spinal cord injury [21-22]. A placebo-controlled phase II trial demonstrated that over a four-week period, 40.3% compared to 23.4% of patients with opioid-induced bowel dysfunction (OIBD) achieved an increase of ≥1 spontaneous bowel movement per week from baseline with prucalopride (4 mg) as compared to placebo, respectively (p = 0.002) [22].

Prucalopride is also effective in reducing post-operative complications. A phase II randomized clinical trial was conducted on 110 post-op patients who had undergone elective gastrointestinal surgery. Fifty-five patients were treated with oral prucalopride (2 mg/day) and the other 55 were given placebo. Patients treated with prucalopride had a shorter time to defecation (65.0 vs. 94.5 h, p = 0.001), passage of flatus (53.0 vs. 73.0 h, p < 0.001), and postoperative length of stay (7.0 vs. 8.0 days, p = 0.001) than controls [23].

Prucalopride can be used for the purpose of bowel cleansing before colonoscopy. Corleto et al. demonstrated that the colon cleansing was optimal in 87% of the patients who were treated with combined prucalopride and 1L polyethylene glycol (PEG) solution instead of the 2L which is normally used. Two patients that used prucalopride reported mild headaches. Therefore, prucalopride can be used for bowel preparation in patients who are unable to drink large quantities of PEG solution [24].

Cinca et al. compared prucalopride with polyethylene glycol-3350 with electrolytes (PEG 3350+E), an osmotic laxative. Two groups of 120 patients each were given prucalopride and PEG 3350+E respectively for four weeks. In the per-protocol population, 66.67% patients in the PEG 3350+E group compared to 56.52% in the prucalopride group achieved more than three SCBMS per week. In the modified intention to treat population, similar results were found but there was more improvement in abdominal symptoms with prucalopride after the first week of treatment. The limiting factor was the length of the study which was only four weeks. Further studies have to be done to determine the long-term efficacy of both therapies [25].

Although many treatment options are common for both constipation-predominant IBS (IBS-C) and CIC like lubiprostone, linaclotide and the older 5-hydroxytryptamine receptor 4 (5-HT4) agonists (tegaserod), some therapies have shown a better response in one group as compared to other. For instance, IBS-C patients respond well to treatments that are specific to pain like antidepressants and cognitive behavioral therapy, whereas prucalopride and pelvic floor biofeedback has more efficacy in CIC group [26].

Prucalopride has shown efficacy in patients with systemic sclerosis (SSc) intestinal disease. In a study by Vigone et al. including 40 patients, 29 participants completed the study. Prucalopride was significantly associated with more intestinal evacuations (p < 0.001) and improvements in reflux (p < 0.005) and bloating (p = 0.01) scores [27].

Prucalopride has also shown efficacy in pediatric constipation. Cisapride, a 5-HT4 receptor agonist was previously approved for childhood constipation, but due to its implication in serious cardiac adverse effects such as arrythmias and sudden death, it was withdrawn. Children given a single dose of prucalopride 0.03 mg/kg (approximately equivalent to a 2 mg dose in an adult weighing 70 kg) had identical pharmacokinetics as studied in adults except for a lower systemic clearance. Children with CIC when treated with prucalopride over eight weeks had a mean frequency of 6.8 bowel movements per week, improvement in stool consistency, and a decrease in frequency of fecal incontinence [28].

In Canada, prucalopride has been approved for the treatment of female patients with chronic idiopathic constipation who have failed therapy with at least two laxatives from different classes over a six-month period. It is the only agent that is recommended by the National Institute for Health Care Excellence (NICE) for chronic constipation in women [29]. Table 1 below shows the adverse effects of prucalopride compared to other drugs used in chronic constipation, and Table 2 below shows the efficacy of prucalopride and other drugs used in chronic constipation.

**Table 1:** Comparison of adverse effects of prucalopride with other drugs used in chronic constipation

Drugs	Adverse Effects	Reference number
Prucalopride	Abdominal pain, diarrhea, nausea, and headache	[14]
Velusetrag	Diarrhea, headache, nausea, vomiting, flatulence, and abdominal pain	[30]
Lubiprostone	Vomiting, nausea, and abdominal cramping	[31]
Linaclotide	Diarrhea	[32]
Plecanatide	Diarrhea, sinusitis, upper respiratory tract infection, abdominal distension, flatulence, abdominal tenderness, and increased levels on liver biochemical tests	[33]

**Table 2:** Efficacy of prucalopride and other drugs used in chronic constipation in terms of CSBM and SBM CSBM; Complete spontaneous bowel movements, SBM; Short bowel movements

Drugs	CSBM	SBM	Reference number
Prucalopride	1.4	4.9	[22]
Velusetrag	2	3.5	[30]
Lubiprostone	2.6	3.2	[34, 35]
Linaclotide	1.8	4.8	[36, 37]
Plecanatide	2.2	3.1	[38]

## Conclusions

Prucalopride is a very useful alternate agent in patients with chronic constipation who have failed laxative therapy and has also been approved for use in Canada. Due to its highly selective action on 5-HT4 serotonin receptor, it is very well-tolerated with fewer cardiac adverse effects. However, randomized prospective trials involving larger populations are needed to further explore the efficacy and safety profile of prucalopride.

## References

[1] Lacy BE, Mearin F, Chang L (2016). Bowel disorders. Gastroenterology.

[2] Grundy D, Al-Chaer ED, Aziz Q (2006). Fundamentals of neurogastroenterology: basic science. Gastroenterology.

[3] Gallegos-Orozco JF, Foxx-Orenstein AE, Sterler SM, Stoa JM (2012 ). Chronic constipation in the elderly. Am J Gastroenterol.

[4] Bassotti G, Chistolini F, Sietchiping-Nzepa F, De Roberto G, Morelli A, Chiarioni G (2004). Biofeedback for pelvic floor dysfunction in constipation. BMJ.

[5] Everhart JE, Go VL, Johannes RS, Fitzsimmons SC, Roth HP, White LR (1989). A longitudinal survey of self-reported bowel habits in the United States. Dig Dis Sci.

[6] Wald A, Scarpignato C, Mueller-Lissner S (2008). A multina­tional survey of prevalence and patterns of laxative use among adults with self-defined constipation. Aliment Pharmacol Ther.

[7] Tse Y, Armstrong D, Andrews CN (2017). Treatment algorithm for chronic idiopathic constipation and constipation-predominant irritable bowel syndrome derived from a Canadian national survey and needs assessment on choices of therapeutic agents. Can J Gastroenterol Hepatol.

[8] (2019). Drug approval package: Motegrity (prucalopride). https://www.accessdata.fda.gov/drugsatfda_docs/nda/2018/210166Orig1s000TOC.cfm.

[9] Potet F, Bouyssou T, Escande D, Baró I (2001). Gastrointestinal prokinetic drugs have different affinity for the human cardiac human ether-à-gogo K+ channel. J Pharmacol Exp Ther.

[10] De Maeyer JH, Lefebvre RA, Schuurkes JA (2008). 5‐HT4 receptor agonists: similar but not the same. Neurogastroenterol Motil.

[11] Bouras EP, Camilleri M, Burton DD, Thomforde G, McKinzie S, Zinsmeister AR (2001). Prucalopride accelerates gastrointestinal and colonic transit in patients with constipation without a rectal evacuation disorder. Gastroenterology.

[12] Rao SS, Rattanakovit K, Patcharatrakul T (2016). Diagnosis and management of chronic constipation in adults. Nat Rev Gastroenterol Hepatol.

[13] Quigley EM, Vandeplassche L, Kerstens R, Ausma J (2009). Clinical trial: the efficacy, impact on quality of life, and safety and tolerability of prucalopride in severe chronic constipation--a 12-week, randomized, double-blind, placebo-controlled study. Aliment Pharmacol Ther.

[14] Camilleri M, Van Outryve MJ, Beyens G, Kerstens R, Robinson P, Vandeplassche L (2010). Clinical trial: the efficacy of open label prucalopride treatment in patients with chronic constipation - follow-up of patients from the pivotal studies. Aliment Pharmacol Ther.

[15] Yiannakou Y, Piessevaux H, Bouchoucha M (2015). A randomized, doubleblind, placebo controlled, phase 3 trial to evaluate the efficacy, safety, and tolerability of prucalopride in men with chronic constipation. Am J Gastroenterol.

[16] Camilleri M, Piessevaux H, YiannakouY YiannakouY (2016). Efficacy and safety of prucalopride in chronic constipation: an integrated analysis of six randomized, controlled clinical trials. Dig Dis Sci.

[17] Camilleri M, Beyens G, Kerstens R, Robinson P, Vandeplassche L (2009). Safety assessment of prucalopride in elderly patients with constipation: a double-blind, placebo-controlled study. Neurogastroenterol Motil.

[18] Smith WB, Mannaert E, Verhaeghe T, Kerstens R, Vandeplassche L, and de Velde VV (2012). Effect of renal impairment on the pharmacokinetics of prucalopride: a single-dose open-label phase I study. Drug Des Devel Ther.

[19] Mearin F, Ciriza C, Mínguez M (2016). Clinical practice guideline: irritable bowel syndrome with constipation and functional constipation in the adult. Rev Esp Enferm Dig.

[20] Bellini M, Usai-Satta P, Bove A (2017 Dec). Chronic constipation diagnosis and treatment evaluation: the “CHRO. CO. DI. TE” study. BMC Gastroenterol.

[21] Krogh K, Jensen MB, Gandrup P (2002). Efficacy and tolerability of prucalopride in patients with constipation due to spinal cord injury. Scand J Gastroenterol.

[22] Sloots CE, Rykx A, Cools M, Kerstens R, De Pauw M (2010). Efficacy and safety of prucalopride in patients with chronic noncancer pain suffering from opioid-induced constipation. Dig Dis Sci.

[23] Gong J, Xie Z, Zhang T (2016). Randomised clinical trial: prucalopride, a colonic pro-motility agent, reduces the duration of post-operative ileus after elective gastrointestinal surgery. Aliment Pharmacol Ther.

[24] Corleto VD, Antonelli G, Coluccio C (2017). Efficacy of prucalopride in bowel cleansing before colonoscopy: results of a pilot study. World J Gastrointest Endosc.

[25] Cinca R, Chera D, Gruss HJ, Halphen M (2013). Randomised clinical trial: macrogol/PEG 3350+electrolytes versus prucalopride in the treatment of chronic constipation - a comparison in a controlled environment. Aliment Pharmacol Ther.

[26] Siah KT, Wong RK, Whitehead WE (2016 Mar). Chronic constipation and constipation-predominant IBS: separate and distinct disorders or a spectrum of disease?. Gastroenterol Hepatol (N Y).

[27] Vigone B, Caronni M, Severino A (2017). Preliminary safety and efficacy profile of prucalopride in the treatment of systemic sclerosis (SSc)-related intestinal involvement: results from the open label cross-over PROGASS study. Arthritis Res Ther.

[28] Winter HS, Di Lorenzo C, Benninga MA (2013). Oral prucalopride in children with functional constipation. J Pediatr Gastroenterol Nutr.

[29] Pennant M, Orlando R, Barton P, Bayliss S, Routh K, Meads C (2011). Prucalopride for the treatment of women with chronic constipation in whom standard laxative regimens have failed to provide adequate relief. Health Technol Assess.

[30] Goldberg M, Li YP, Johanson JF (2010). Clinical trial: the efficacy and tolerabilityof velusetrag, a selective 5-HT4agonist with high intrinsic activity, in chronic idiopathic constipation - a 4-week, randomized, double-blind, placebo-controlled, dose-response study. Aliment Pharmacol Ther.

[31] Lacy BE, Levy LC (2008). Lubiprostone: a novel treatment for chronic constipation. Clin Interv Aging.

[32] Thomas RH, Allmond K (2013). Linaclotide (Linzess) for irritable bowel syndrome with constipation and for chronic idiopathic constipation. P T.

[33] (2019). TRULANCE (plecanatide) tablets - FDA. https://www.accessdata.fda.gov/drugsatfda_docs/label/2018/208745s001lbl.pdf.

[34] Pennington B, Marriott ER, Lichtlen P, Akbar A, Hatswell AJ (2018). The cost-effectiveness of lubiprostone in chronic idiopathic constipation. Pharmacoecon Open.

[35] Jamal MM, Adams AB, Jansen JP (2015). A randomized, placebo-controlled trial of lubiprostone for opioid-induced constipation in chronic noncancer pain. Am J Gastroenterol.

[36] Schoenfeld P, Lacy BE, Chey WD (2017). Low-dose linaclotide (72 μg) for chronic idiopathic constipation: a 12-week, randomized, double-blind, placebo-controlled trial. Am J Gastroenterol.

[37] Lembo A, Johnston J, MacDougall J (2008). Linaclotide significantly improved bowel habits and relieved abdominal symptoms in adults with chronic constipation: data from a large 4-week, randomized, double-blind, placebo-controlled study. Gastroenterology.

[38] DeMicco M, Barrow L, Hickey B (2017). Randomized clinical trial: efficacy and safety of plecanatide in the treatment of chronic idiopathic constipation. Therap Adv Gastroenterol.

